# Not What It Seems: Deep Tissue Infection Presenting as Cellulitis

**DOI:** 10.5811/westjem.2015.6.27609

**Published:** 2015-10-20

**Authors:** Caroline T. Brandon, Tarina Kang

**Affiliations:** Los Angeles County Medical Center+University of Southern California Medical Center, Department of Emergency Medicine, Los Angeles, California

A 34-year-old male with diabetes presented to the emergency department with four days of progressively worsening redness, swelling and pain to his left buttock. The patient denied fevers, chills, rectal pain or purulent drainage from his rectum. His initial vital signs were heart rate of 82; blood pressure of 146/92; and temperature of 98.2°F. The left buttock had a poorly circumscribed area of induration; however, there was no fluctuance or crepitace. Rectal exam was unremarkable. Because the patient’s buttock pain was disproportionate to his exam findings, a point-of-care ultrasound was performed to determine if a more extensive process was present. The ultrasound demonstrated cobblestoning, fascial thickening with edema, and a large 4.5cm fluid collection extending and adjacent to the rectum (Figure 1). A computed tomography (CT) of the pelvis with IV contrast confirmed the presence of a perirectal abscess (Figure 2).

Perirectal abscesses and fistulas are common in adults. Abscess formation begins as an infection of the anal glands. Over time, this infection can spread to surrounding tissues leading to fistula formation in roughly 40% of patients. The ratio of occurrence in males to females in the adult population is 2:1. Diagnosis of a peri-rectal abscess is clinical; however, ultrasound and CT can be used in indefinite cases. Treatment requires surgical incision and drainage with packing and follow up. Reoccurrence occurs in roughly 10% of patients. The patient was seen by a surgeon who performed a bedside incision and drainage, and the patient was started on IV antibiotics and discharged the next day.

## Figures and Tables

**Figure 1:**
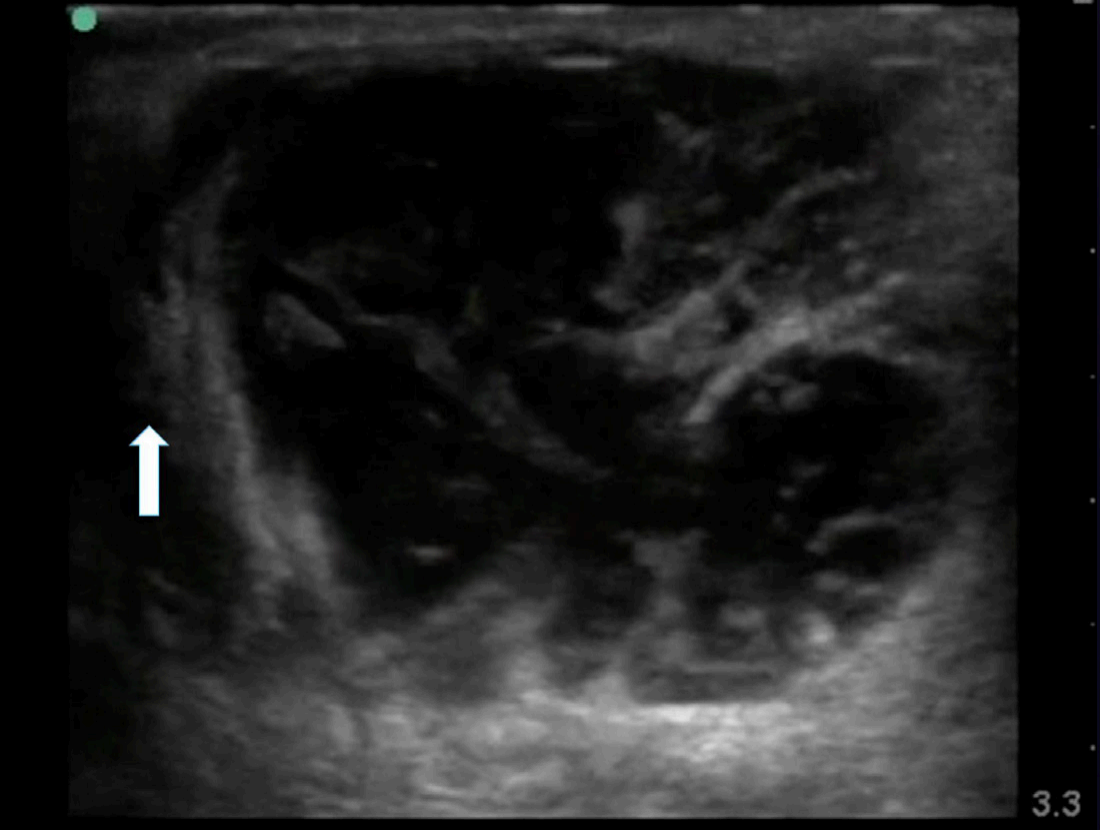
Large fluid collection adjacent to the rectum (white arrow).

**Figure 2:**
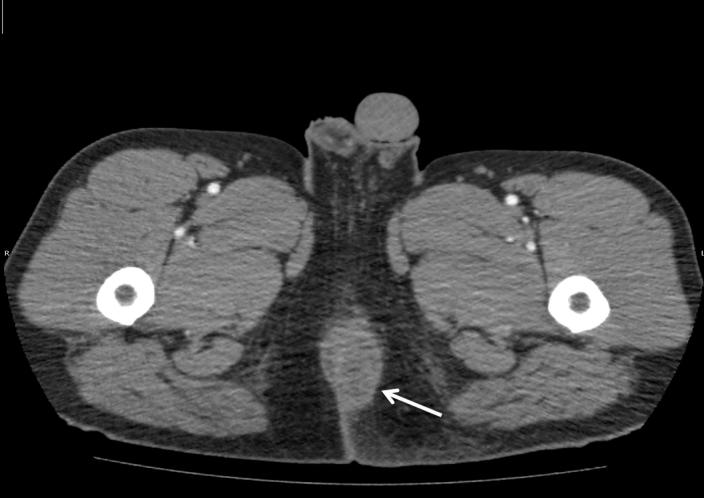
Pelvic CT showing peri-rectal fluid collection (white arrow).

